# Exo- and endoscopic two-step approach for meningeal tumours invading the lateral wall of large dural venous sinuses: how I do it

**DOI:** 10.1007/s00701-024-06298-2

**Published:** 2024-10-07

**Authors:** Kenichiro Iwami, Tadashi Watanabe, Kazuhito Takeuchi, Ryuta Saito

**Affiliations:** 1 Department of Neurosurgery, Nagoya University Graduate School of Medicine, Nagoya, Japan; 2 Department of Neurosurgery, Aichi Medical University, Aichi, Japan

**Keywords:** Meningeal tumour, Dural venous sinus, Exoscope, Endoscope

## Abstract

**Background:**

Treating meningeal tumours invading the large dural venous sinuses is a subject of debate regarding the approach for removing the intra-sinus components. Additionally, directly observing the invasion site of tumours invading the lateral wall of the sinus is difficult.

**Method:**

We describe our exo- and endoscopic two-step approach (EETA): an exoscope is used to remove the extra-sinus component, while an endoscope is used to observe the invaded lateral wall and remove the intra-sinus component.

**Conclusion:**

EETA can be a viable option for treating meningeal tumours invading the venous sinus owing to its high resection rate and low invasiveness.

**Supplementary Information:**

The online version contains supplementary material available at 10.1007/s00701-024-06298-2.

## Relevant surgical anatomy

Sindou et al. reported that 18% of all meningiomas affect the large dural venous sinuses (LDVS) [5, 7]. Likewise, Yip et al. reported that 57.1% of all solitary fibrous tumours (SFT) involved the LDVS [8]. Meningeal tumours frequently invade the lateral wall of the LDVS; accordingly, the grading systems used are based on the degree of invasion, ranging from attachment to the external surface of the sinus wall to complete sinus occlusion [6]. In such cases, observing the invaded sinus wall (sinus orifice) or intra-sinus tumour without performing an additional incision of the sinus wall is relatively difficult. However, accessing tumour-obstructed sinuses by incising the sinus wall may increase the risk of sacrificing the collateral veins [3]. Close observation of the sinus orifice and surrounding bridging veins and removal of the intra-sinus component, while preserving as much of the normal sinus wall as possible, can help improve tumour removal rates and avoid venous outflow obstruction.

## Description of the technique

**Fig. 1:**
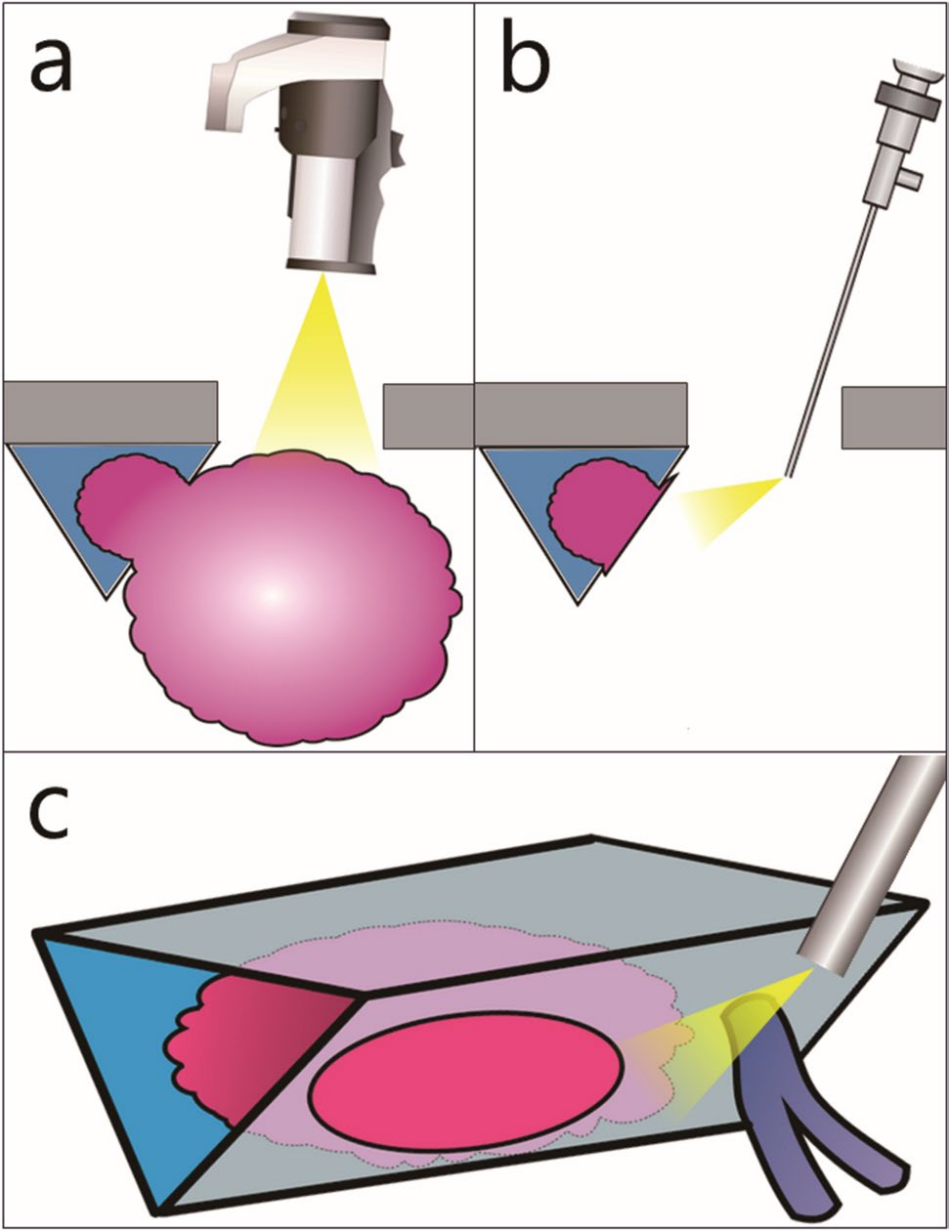
Schematic of EETA. **a** The first step of EETA. The extra-sinus component is removed using an exoscope **(b)** The second step of EETA. The intra-sinus component is removed using an endoscope. **c** The endoscope provides clear views of the intra-sinus tumour and surrounding venous structures, including the sinus wall and bridging veins

The exo- and endoscopic two-step approach (EETA) for meningeal tumours invading the LDVS comprises two steps. In the first step, the tumour is bisected into the intra- and extra-sinus at the site of the LDVS invasion, and the extra-sinus component is removed using an exoscope (ORBEYE; Olympus, Tokyo, Japan) using the same technique as that in microscopic surgery (Fig. 1a). In the second step, the sinus orifice is observed in detail using rigid endoscopes (0°, 30°, 45°, or 70°; outer diameter, 4 mm; Karl Storz, Tuttlingen, Germany, or Olympus, Tokyo, Japan), and the intra-sinus component is removed while preserving as much of the normal venous wall as possible (Fig. 1b). The basic principle is to remove the intra-sinus tumour while avoiding any new obstruction of venous outflow. 1) In cases without complete sinus occlusion (partial invasion), the sinus orifice is closed by suturing or patching depending on its size following tumour removal [2], whereas 2) in cases with complete sinus occlusion (complete invasion), bleeding occurring from the distal and proximal ends of the sinus after tumour removal is stopped by suturing the vessel wall or filling the sinus lumen with fibrin glue-soaked haemostatic fabric. The endoscope provides a clear view of the intra-sinus tumour and surrounding venous structures, therefore, enabling venous outflow preservation depending on the case (Fig. 1c). The EETA procedure in four representative cases is given below:

### Case 1: Tentorial meningioma invading the right transverse sinus (Fig. 2, Video 1)

**Fig. 2:**
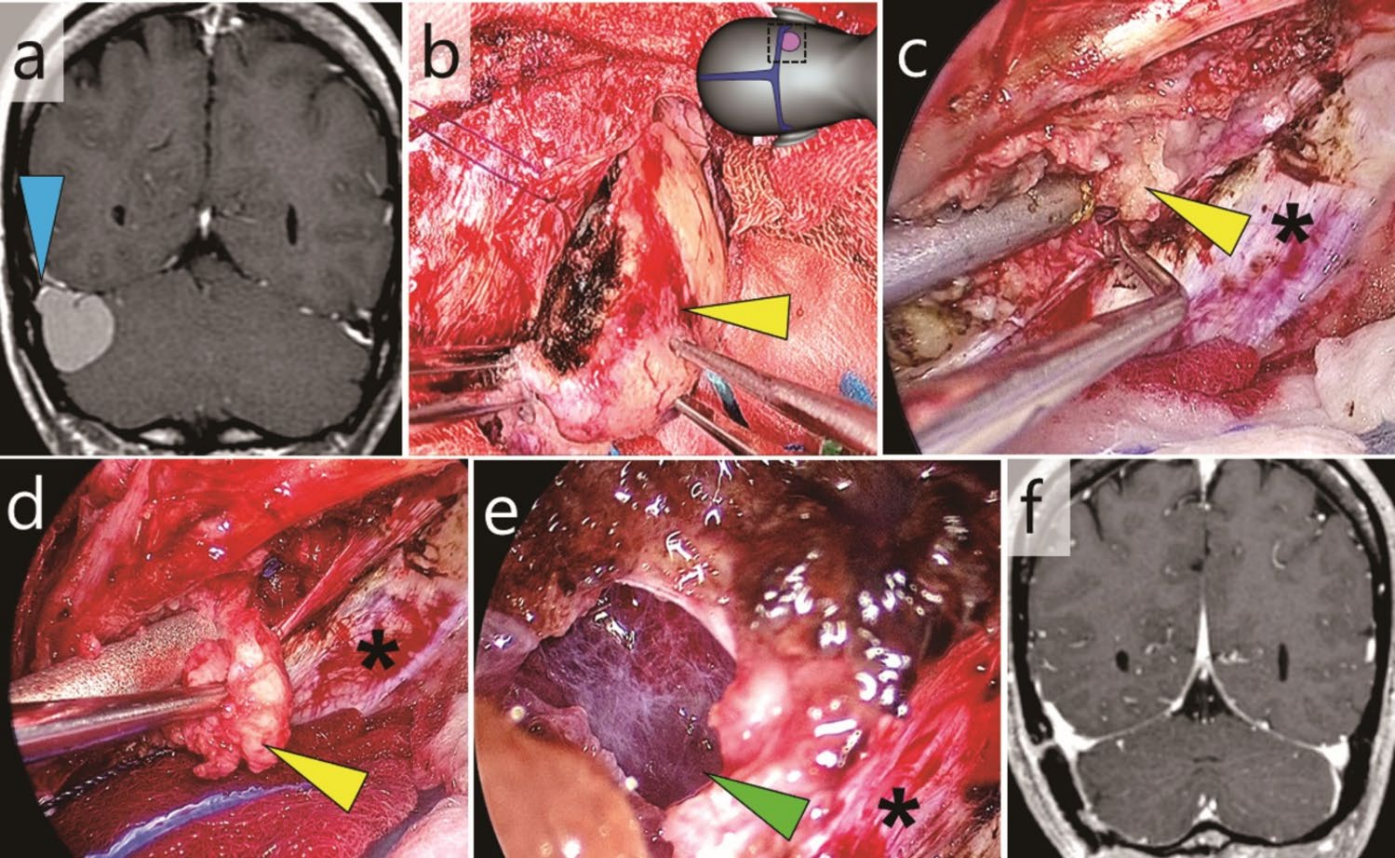
Case 1: Tentorial meningioma with partial invasion into the transverse sinus. a Pre-operative gadolinium-enhanced T1-weighted magnetic resonance image (MRI) (coronal); venous blood flow remained cranially within the sinus (blue arrowhead). b The extra-sinus component (yellow arrowhead) was exoscopically removed in an en bloc fashion. The illustration on the top right indicates the field of view. c Observation of the sinus orifice on the inferolateral wall of the transverse sinus using an endoscope. Yellow arrowhead, intra-sinus component; *, tent (d) The intra-sinus component (yellow arrowhead) was endoscopically removed. *, tent (e) The intra-sinus tumour was removed while preserving the thin vessel wall (green arrowhead). f Post-operative gadolinium-enhanced T1-weighted MRI (coronal)

This was a case of tentorial meningioma with partial invasion of the transverse sinus. The sinus orifice was in the inferolateral wall, with residual blood flow occurring cranially within the transverse sinus. The tumour was excised through a small suboccipital craniotomy while preserving the sinus blood flow.

### Case 2: Torcular meningioma invading the confluence of sinuses (Fig. 3, Video 2)

**Fig. 3:**
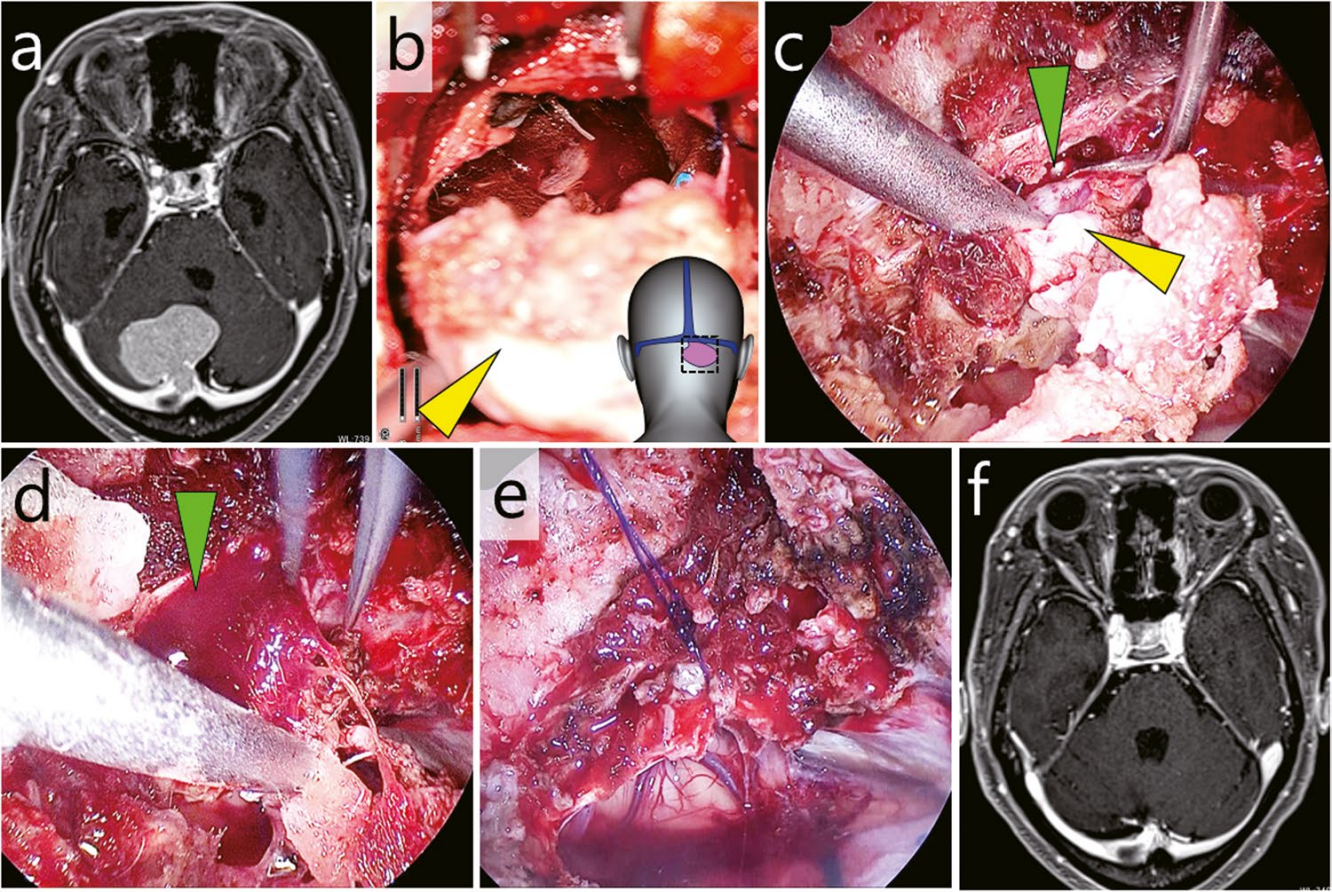
Case 2: Torcular meningioma with partial invasion into the confluence of sinuses. **a** Pre-operative gadolinium-enhanced T1-weighted MRI (**b**) The extra-sinus component (yellow arrowhead) was exoscopically removed. The illustration on the bottom right indicates the field of view. **c** The intra-sinus component (yellow arrowhead) was endoscopically removed. Green arrowhead, sinus orifice (**d**) Following removal of the intra-sinus tumour, venous bleeding occurred from the sinus orifice (green arrowhead). **e** The sinus orifice was closed by suturing. **f** Post-operative gadolinium-enhanced T1-weighted MRI

This was a case of torcular meningioma with partial invasion into the confluence of the sinuses. The tumour was excised through a small suboccipital craniotomy, while preserving the sinus blood flow.

### Case 3: Parasagittal meningioma invading the superior sagittal sinus (Fig. 4, Video 3)

**Fig. 4:**
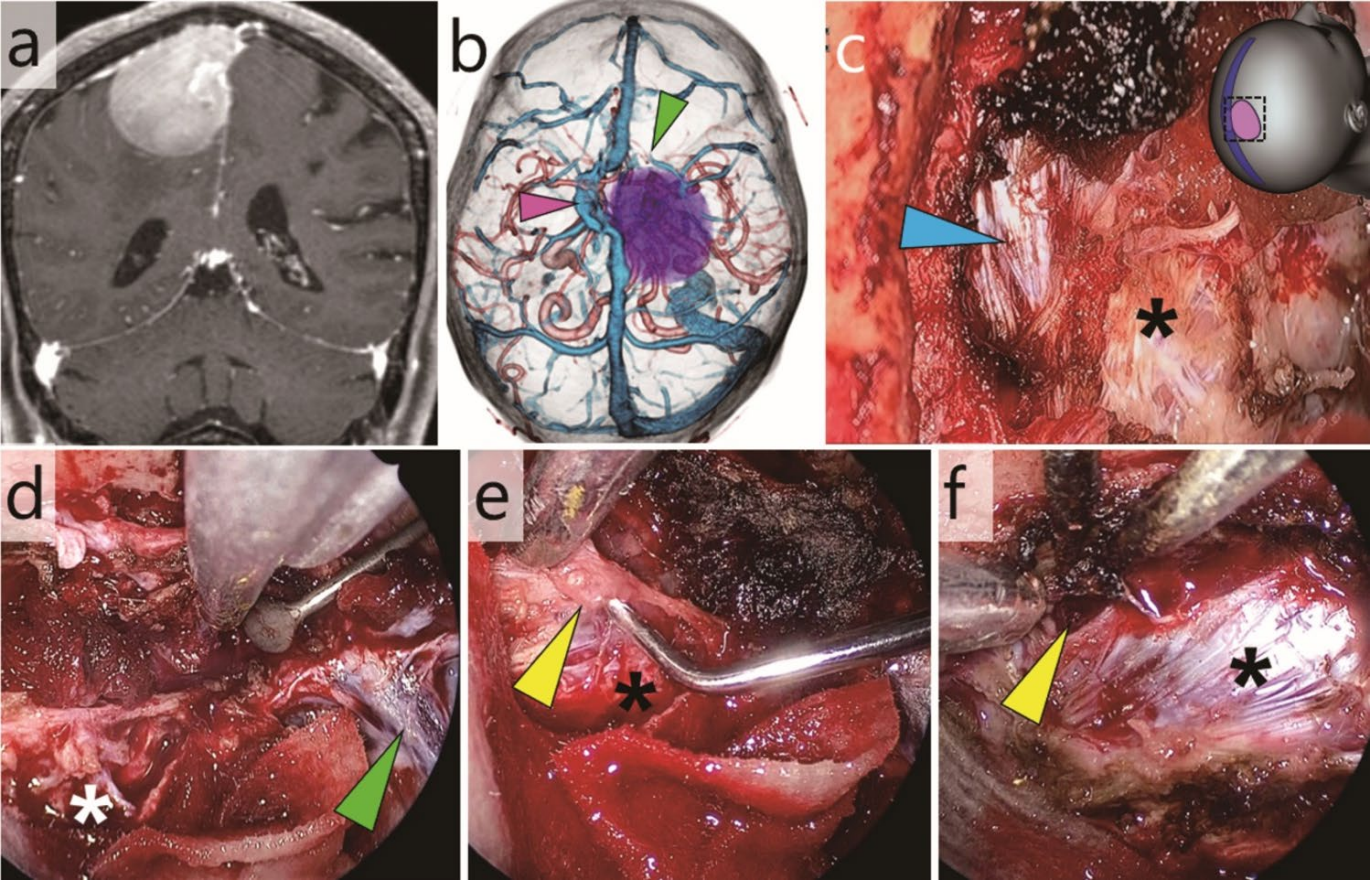
Case 3: Parasagittal meningioma with complete invasion into the superior sagittal sinus. **a** Pre-operative gadolinium-enhanced T1-weighted MRI. **b** Preoperative three-dimensional (3D) computed tomography (CT) revealed a collateral venous flow immediately to the left of the tumour-invaded sinus (pink arrowhead) and a bridging vein located just anterior to the tumour (green arrowhead). **c** The extra-sinus component was exoscopically removed. The illustration on the top right indicates the field of view. Blue arrowhead, sinus orifice; *, falx. **d** The bridging vein located anterior to the sinus orifice was preserved (green arrowhead). *, falx (**e** and **f**) The residual tumour at the posterior edge of the sinus orifice was endoscopically removed. Yellow arrowheads, residual tumour and opened sinus lumen; *, falx

This was a case of a parasagittal meningioma with complete invasion of the superior sagittal sinus. The tumour was excised through a small frontal craniotomy, while preserving the collateral venous flow and bridging veins.

### Case 4: Tentorial SFT invading the straight sinus (Fig. 5, Video 4)

**Fig. 5:**
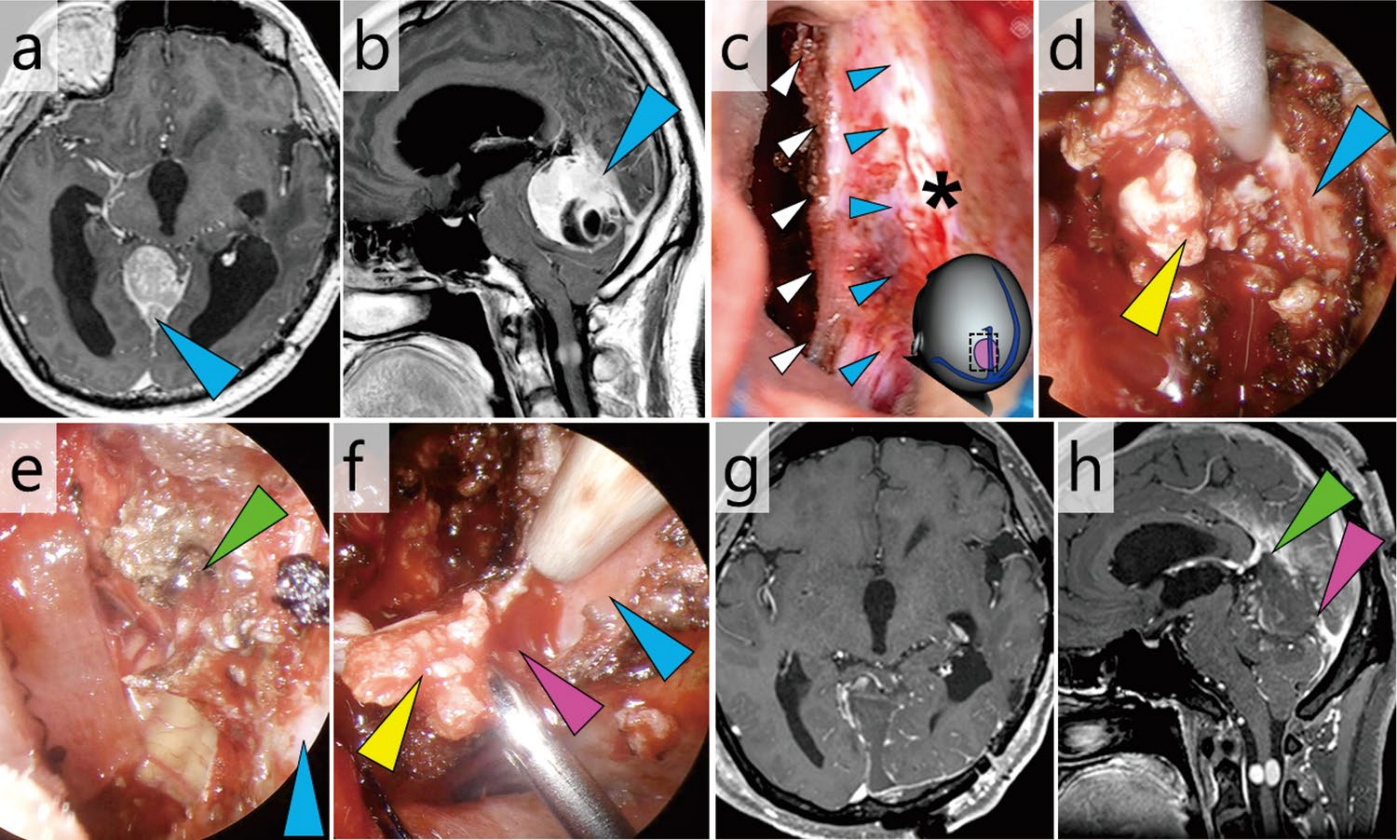
Case 4: Tentorial SFT with complete invasion into the straight sinu. a and b Pre-operative gadolinium-enhanced T1-weighted MRI (**a**, axial; **b**, sagittal). Blue arrowheads, intra-sinus tumour (**c**) Most of the extra-sinus component was exoscopically removed using a left-sided transtentorial approach. The illustration on the bottom right indicates the field of view. Blue arrowhead, tumour-occluded straight sinus; white arrowheads, medial cut end of tentorium; *, falx. **d** The endoscope was inserted into the subtentorial space and the intra-sinus component (yellow arrowhead) was removed from the left side. Blue arrowhead, inner surface of the tumour-obstructed sinus. **e** The residual tumour at the distal edge of the obstructed sinus was endoscopically removed. Green arrowhead, distal end of the sinus after tumour removal; blue arrowhead, inner surface of the tumour-obstructed sinus. **f** The residual tumour at the proximal edge of the obstructed sinus was endoscopically removed. Yellow arrowhead, intra-sinus tumour; pink arrowhead, bleeding from the proximal end of the sinus, blue arrowhead, inner surface of the tumour-obstructed sinus. **g** and **h** Post-operative gadolinium-enhanced T1-weighted MRI (**g**, axial; **h**, sagittal). The venous blood flow was preserved at the distal (green arrowhead) and proximal (pink arrowhead) ends of the sinus

This was a case of a tentorial SFT with complete invasion of the straight sinus. The tumour was excised using a left-sided transtentorial approach. Following tumour removal, the straight sinus was closed proximally and distally, ensuring the venous blood flow of the torcula and vein of Galen.

Although an exoscope can be replaced by a microscope, using an endoscope is preferable for observing the lateral aspect of LDVS. The reasons for using an exoscope are as follows: 1) the same surgical monitor is used for the exoscope and endoscope; therefore, switching between them is easy; 2) it allows for 3D image sharing; and 3) it is ergonomically superior, allowing the surgeon to view a wide area through a small craniotomy and perform surgery in a comfortable posture [1, 4].

## Indications

EETA is used for meningeal tumours invading the lateral wall of the LDVS owing to its high resection rate and low invasiveness. The main indications are parasagittal, falcine, or tentorial meningiomas, which often invade the lateral wall of the superior sagittal or transverse sinus. Because of the difficulty in performing complex suturing procedures under endoscopy, EETA is well suited for cases in which reconstruction of the sinus is not necessary or where the sinus orifice can be closed with relatively simple procedures, including patching or limited suturing.

## Limitations

As EETA is not suitable for larger tumours accompanied by severe brain oedema or venous congestion, a larger craniotomy or venous reconstruction approach should be considered. In cases where tumours involve the superior wall of the LDVS, intra-sinus tumours can be approached using a microscope or exoscope, without requiring an endoscope. As complex suturing techniques are difficult to perform under endoscopy, EETA is not suitable for cases requiring many sutures. The decision and approach for removing the intra-sinus components in meningeal tumours invading the LDVS have been the subject of debate. Although EETA is a new surgical option, it does not provide an absolute solution to this debate. We must continue to carefully determine treatment plans on a case-by-case basis.

## How to avoid complications

Careful pre-operative assessment of venous outflow, not only around the tumour but also throughout the brain, helps to prevent inadvertent surgical bleeding and new obstructions to the venous outflow. While contrast-enhanced CT may be sufficient, angiography is preferred. Body and head positions should be adjusted to prevent excessive bleeding and air embolism associated with LDVS opening. When closing the sinus stump, avoiding the occlusion of branches or collateral veins is crucial. The reconstruction method for closing the sinus orifices while maintaining the sinus blood flow, including patching or suturing, should be selected based on the orifice size [2].

## Specific information for the patient

General surgical risks such as infection and bleeding, exist. LDVS opening can result in excessive bleeding and air embolism, while post-operative venous congestion may occur due to venous outflow obstruction. In some patients, un-resectable tumours remaining around the LDVS may require radiation therapy.

## Supplementary Information

Below is the link to the electronic supplementary material.

Supplementary file1 (MP4 54024 KB)

Supplementary file2 (MP4 120726 KB)

Supplementary file3 (MP4 51113 KB)

Supplementary file4 (MP4 103077 KB)

## Data Availability

No datasets were generated or analysed during the current study.

## Abbreviations

EETA: Exo- and endoscopic two-step approach
LDVS: Large dural venous sinus
SFT: Solitary fibrous tumour
MRI: Magnetic resonance imaging
3D: Three-dimensional
CT: Computed tomography
